# Effect of dawri‐damaa (*Pentas schimperiana*) leaf meal supplementation on performances, carcass characteristics, and economic feasibility of sheep fed native grass hay

**DOI:** 10.1002/fsn3.4156

**Published:** 2024-04-08

**Authors:** Gubil Bekele Dillo, Taye Tolemariam, Asrat Guja Amejo

**Affiliations:** ^1^ Department of Animal Science, College of Agriculture and Veterinary Medicine Jimma University Jimma Ethiopia; ^2^ Department of Animal Science, College of Agriculture Sciences Arba Minch University Arba Minch Ethiopia

**Keywords:** carcass, dawri‐damaa, economic feasibility, sheep

## Abstract

With a growing export and local market, sheep farming is critical to the economy of smallholder farmers; however, indigenous sheep breeds usually exhibit low carcass output and live weight due to nutritional constraints. The study aimed to investigate the impact of supplementing local sheep with dawri‐damaa leaf meal (DDLM) on their performance, carcass characteristics, and profitability when fed native grass hay. The research used a randomized complete block design (RCBD) to administer four feeding treatments (T1, T2, T3, and T4 at 0%, 30%, 50%, and 70% DDLM, respectively) to 20 local uncastrated male lambs having an average 23.72 ± 0.73 kg body weight and average age of 11.72 ± 0.74 months. Greater doses of DDLM in the diet resulted in greater average daily gain (ADG) of 51.4–83.8 g/day and feed conversion efficiency (FCE) of 0.066–0.089 in lambs, compared to 15.0 g/day ADG and 0.025 FCE in lambs under control diet (T1). Additionally, the lambs had increased slaughter body weight (SBW), empty body weight (EBW), and hot carcass weight (HCW) in T4 and T3 compared to other treatments (*p* < .05). There were no significant differences in dressing percentages (DPs) across any treatment group. The study also discussed the economic viability of supplementing with DDLM, suggesting that feeding 70% daily dry matter of DDLM at 2.5% live weight might be beneficial depending on availability, access, and cost factors. In conclusion, feeding DDLM up to 70% has improved the performance of sheep and is economically feasible. Further research might be required to discover whether such levels of inclusion are harmful and apply different processing methods for feeding animals.

## Graphical abstract

The study found that supplementing sheep with dawri‐damma leaf meal (DDLM) resulted in increased body weight, average daily gain, and feed conversion rate. The three doses of DDLM supplementation (30%, 50%, and 70%) resulted in daily increases of 51.4 g, 68.0 g, and 83.8 g, respectively, which outperformed the national meat production average of 50.0 g per animal per day (Melaku and Betsha, *Tropical Animal Health and Production*, 40, 2008 and 553) and were significantly higher than those observed in lambs fed a basal diet (T1) of 15.0 g in this study. Trends in body weight changes of local growing lambs in Dawuro fed native grass hay supplemented with different levels of DDLM during a 90‐day feeding period.

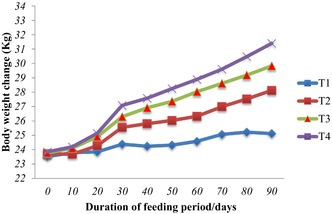

## 1 INTRODUCTION

The global demand for sheep meat is rising, particularly in emerging countries, which are anticipated to lead the majority of sheep production (Chikwanha et al., 2018; FAO, 2011; Lage et al., 2020). In developing countries, the increased meat consumption is linked to lower prices (Delgado, 2003). However, it's crucial to recognize that consumer behavior and preferences significantly shape the perceived quality of meat, often outweighing price considerations (Henchion et al., 2014).

Globalization is reshaping the lamb meat market, influenced not only by customs and traditions but also by increasing consumer awareness regarding food product origins and associated quality aspects (Erasmus et al., 2017). While sheep raised in feedlots often show improved carcass weight, there is a noted decrease in meat quality (Hou et al., 2023; Luo et al., 2019; Wang et al., 2018). Optimal use of grasslands is an effective means of converting energy into food, making grazing systems a pivotal component in the rapidly growing global lamb meat production. Ethiopia, home to the second‐largest sheep population in Africa, primarily relies on natural grassland grazing within its production system, which is low in nutritional value.

Ethiopia's sheep breeds are highly sought‐after in the Middle East due to their taste and the organic nature of their production (Animut & Wamatu, 2014). The primary destinations for Ethiopia's sheep and goat meat exports include the United Arab Emirates, Saudi Arabia, Qatar, Oman, and Kuwait (OEC, 2021; USAID, 2021). In 2021, Ethiopia recorded $93.9 million in sheep and goat meat exports, ranking as the 10th largest exporter of sheep and goat meat globally, and marking it as the 9th most exported product from Ethiopia in the same year (OEC, 2021).

Local sheep typically demonstrate poor carcass output and limited live weight gain, averaging 50 g per animal daily for meat production (Melaku & Betsha, 2008). Moreover, the average lamb carcass weight of 10 kg falls below the global average of 15.8 kg (FAO, 2013). Nutrition plays a crucial role in addressing these challenges, as it is considered one of the most difficult production elements contributing to the subsector's low output (Getahun, 2015). Moreover, the scarcity of feed resources during the dry season and the high prices for the limited available items jeopardize the economic viability of production systems (de Oliveira et al., 2018; Tikam et al., 2015).

Effective husbandry involves planning feed requirements for livestock farms, especially in traditional livestock production systems. Traditional methods lack precision and efficacy, and fundamental information is often unavailable or estimations are rough (Mekonnen et al., 2021). Feed deficiency is a common challenge, and animal weight loss is a common issue.

Increasing livestock output can be accomplished by supplementing locally available feedstuffs with graded quality concentrate feed, reducing scarcity through the introduction of improved local and hybrid forage grasses and legumes. The pursuit of geographically appropriate, less expensive, and more readily available components is one technique for producing carcasses with a differentiated quality pattern (de Oliveira et al., 2018). When compared to other animal products, ruminant meat production has a significant environmental cost, however, attempts to reduce the effect while increasing animal productivity have resulted in solutions (Salami et al., 2019). Farmers and the environment benefit from less feed required for the same or higher yield, demonstrating a clear advantage for livestock breeding and the planet (Gurgeira et al., 2022).

Traditional livestock production techniques typically rely on locally available resources with minimal opportunity costs. *Pentas schimperiana*, also known by the local name Dawri‐damma, belongs to the Rubiaceae plant family. Its leaves and twigs provided animal feed, traditional medicinal value, and a source of income to the Dawuro community (Andarge et al., 2015; Tonamo et al., 2015; Woretaw et al., 2022). Feeding *P. schimperiana* to livestock is a popular nutritional technique in the community for increasing milk yield, improving carcass and meat quality, and benefiting the animal. The plant is utilized in supplementing milk cows, dry cows, heifers, calves, oxen, bulls, sheep, goats, and equines. The study found that *P. schimperiana* might be used as supplementary feeds to enhance the utilization of low‐quality feed resources and improve the performance of ruminants during the dry season when feed is scarce (Woretaw et al., 2022).

This study aimed to assess the influence of dawri‐damaa leaf meal (DDLM) supplementation on feed efficiency, average daily gain, carcass characteristics, and the economic viability of local sheep fed hay grass in the Dawuro Zone of Southwestern Ethiopia.

## 2 MATERIALS AND METHODS

### 2.1 Study area

The study was conducted in the Mareka District of Dawuro Zone, Ethiopia, which is part of the Southern Nations, Nationalities, and Peoples’ Region (SNNPR) state. The zone is bordered by the Gojeb and Omo rivers and is approximately 507 km from Addis Ababa. The field trial site was chosen in Waka Iyesus peasant administration, located 2 km west of Waka and 19 km southwest of Tercha. The annual mean minimum temperature in the zone is 14.9–26.4°C, with an annual mean rainfall range of 1200–1800 mm. The trial site is 2357 m above sea level and located at 70 8′ N latitude and 290 58′ E longitude. Among indigenous browse and legume plants preferred by farmers owning ruminants in the Dawuro Zone, dawri‐damaa, cayshiya, and gasaa have greater nutritional value (Tonamo et al., 2015).

The study found that the dry matter (DM), crude protein (CP), and ash contents, as well as the in vitro DM digestibility, were 43.38%, 14.23%, 3.71%, and 85.18%, respectively (Tonamo et al., 2015). Another study indicated seasonal and agro‐ecological variability of DM and CP contents of *P. schimperiana*. In the highland regions, Woretaw et al. (2022) showed 17.39% CP and 87.41% in vitro DM digestibility levels. However, our unpublished analysis showed that *P. schimperiana* had 90.2% DM, 17.74% CP, and 93.51% organic matter. Moreover, certain phytochemical studies suggested the existence of flavonoids, saponins, steroids, and tannins in *P. schimperiana* (Dinku et al., 2010; Kifle et al., 2022). *Pentas schimperiana* (A. Rich) Vatke (Rubiaceae) is widely used for the treatment of diabetes mellitus and various other ailments in the traditional medical practices of Ethiopia (Dinku et al., 2010). The methanolic leaf extract of *P. schimperiana* holds promise as a potential treatment for diabetes mellitus, especially in resource‐limited settings (Amare et al., 2021). Heba‐tollah et al. (2018) reported that *Pentas* species are commonly used in traditional medicine.

### 2.2 Experimental animals, diets, and feeding

Using the dentition method and owner information, 20 yearling uncastrated male local Dawuro sheep with a mean initial live weight of 23.72 ± 0.73 kg and aged 11.72 ± 0.74 months (mean ± standard error (SE)) were obtained from the local market. The animal studies were approved by the Animal Experimentation Committee and followed the institutional norms of Jimma University's College of Agriculture and Veterinary Medicine. The experiment was carried out in accordance with recommendations made by the European Parliament and the Council of 22 September 2010 on the protection of animals used for scientific purposes. During the study, lambs were housed in pens measuring 1.55 m^2^ each, with a floor area of 1 m^2^, a partition height of 1.4 m, a trough space of 0.12 m for ad libitum diets, and a trough space of 0.25 m for treatment meals. The floor spaces were bedded with dried enset (*Ensete ventricosum*) leaves and replaced on a regular basis based on the conditions. To allow for adaptation, the basal diet and 50 g dawri‐damaa leaf meal (DDLM) were kept for a further seven days before data collection. Experimental feeds were composed of native grass hay as a basal diet and supplements of DDLM and ground maize. The experimental design was randomized complete block design (RCBD), with four treatments, one of which was control (T1). The experimental diets were weighed every morning before being distributed to the individual lambs in separate troughs and offered twice per day (DDLM) at 50% of their daily ration; in the morning and afternoon at 8:30 a.m. and 1:30 p.m., respectively, while ground maize (100 g) was offered once a day at 8:00 a.m. The supplements were adjusted to satisfy the maintenance needs of control lambs and then switched to meet the daily body gain (100 g/day) needs of lambs weighing 20–30 kg (ARC, 1980). Animals from each block were randomly assigned to one of the four treatment groups. The DDLM supplement was given to treatments T1, T2, T3, and T4 at 0%, 30%, 50%, and 70%, respectively, based on daily dry matter intake rate of 2.5% of their live weight (McDonald et al., 2010), and was adjusted as body weight changed. All lambs were offered basal diets ad libitum, and 100 g of ground maize was supplemented to balance energy needs (Table 1). The well‐being of the experimental animals was inspected and recorded twice a day, as specified by the Ethical Committee. The feeding trial lasted 90 days following quarantine and acclimatization to the experimental diet and pen.

**TABLE 1:** Experimental diets allocated for lambs during field trial period.

Treatment	Experimental diets allotted (DM)			Total daily offer (g/day)
	NGH	GM g/day	DDLM g/day	
T_1_ (C)	Ad libitum	100	0	100 g MG + NGH (basal)
T_2_	Ad libitum	100	178 g	T_1_ + 178 g DDLM
T_3_	Ad libitum	100	297 g	T_1_ + 297 g DDLM
T_4_	Ad libitum	100	415 g	T_1_+ 415 g DDLM

Abbreviations: C, control; DDLM, dawri‐damaa leaf meal; DM, dry matter; GM, ground maize; NGH, native grasses hay.

### 2.3 Body weight gain

The study involved taking two consecutive measurements following an overnight fast to ascertain the starting body weights of lambs. Throughout the experiment, the weights were measured at 10‐day intervals. The difference between final body weight and initial body weight was divided by the number of feeding days to get the average daily body weight gain (Abera et al., 2021). Feed conversion efficiency (FCE) was measured by dividing the lambs’ mean daily weight gain by their daily DM consumption (NRC, 1996).

$$\text{Feed conversion efficiency}\;\left(\mathrm{FCE}\right)=\frac{\text{Average daily body weight gain}\;\left(g\right)\;}{\text{Average daily feed intake}\;\left(g\right)\;}$$

### 2.4 Measures of carcass characteristics

The animals were stunned using procedures that caused no unnecessary pain, suffering, discomfort, or long‐term harm to the animals after approval from Jimma University's Ethical Council. The animal was fed the experimental diet, DDLM, at the appropriate level and concentration, with no additives or negative effects introduced. After 90 days of feeding, the sheep were fasted overnight and killed for carcass examination (Merhun et al., 2016). The animals were weighed immediately before slaughter.

The hot carcass weight (HCW) is used to calculate the dressing percentage based on slaughter and empty body weights. After removing the weight of the head, thorax, abdomen, pelvic cavity contents, and legs below the hock and knee joints, the HCW is determined. After severing the vertebrae between the 12th and 13th ribs, the rib‐eye muscle area (REMA) is sketched on a transparent plastic grid and measured (Calnan et al., 2014; Williams, 2002). The sum of the tongue, heart, liver, bile, kidney, empty gut, omental fat, heart fat, and kidney fat is used to compute the proportion of total edible offal components (TEOC). Head without tongue, blood, bladder, lung with esophagus and trachea, stomach content, spleen, skin, and feet are total nonedible offal components (TNOC). By deducting the gut content from the slaughter weight, the empty body weight (EBW) is derived. The dressing percentage (DP) is computed using the Gilmour et al. (1994) formula.

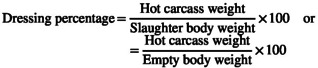




### 2.5 Partial budget analysis

The study investigated the economic efficiency of feeding indigenous sheep in Dawuro, a basic diet of native grass hay, 100 g ground maize, and DDLM at varying levels using the technique indicated by Upton (1979). The research entailed determining the total variable cost (TVC) and return on sale. The purchasing price of each lamb was estimated by dealers from a nearby market, and the selling price was estimated in the same manner before slaughtering took place. The accuracy of weight measurements for sheep was tested in four local marketplaces. Total return (TR) was calculated by subtracting the selling and purchase prices for each lamb. Feed prices were calculated by multiplying the total feed intake for 90 days by market prices since purchase (Fitwi & Tadesse, 2013). The cost of dawri‐damaa leaf obtained from volunteers was also taken into account. When acquiring feed, the current price of feed, transportation and labor costs were all taken into account. Net return (NR) was also computed as the amount of money remaining after subtracting total variable cost (TVC) from total returns (TRs). Similarly, the change in net return (∆NR) was determined by subtracting the change in total return (∆TR) from the change in total variable costs (∆TVC).

NR=TR−TVC/∆NR=∆TR–∆TVC

The percentage marginal rate of return (MRR), which measures the increase in net return (∆NR) associated with each additional unit of expenditure (∆TVC), was calculated as:

$$\mathrm{MRR}\%=\;\frac{∆\mathrm{NR}}{∆\mathrm{TVC}}\times 100\%$$

### 2.6 Statistical analysis

Data were analyzed using the General Linear Model (GLM) procedure of SAS 9.3. Fisher's least significant difference (LSD) test was used to compare treatment means, and the values were considered at 5% probability (Gebrekidan et al., 2019). Therefore, the model employed for data analysis:

$$Y_{\mathrm{ij}}=\mu +t_{i}+b_{j}+e_{\mathrm{ij}};$$

Where: *Y*_*ij*_ = response variable; *μ* = overall mean; *t*_*i*_ = *i*th treatment effect; *b*_*j*_ = *j*th block effect; and *e*_*ij*_ = *ij*th random error.

## 3 RESULTS AND DISCUSSION

### 3.1 Body weight gain and feed conversion efficiency

The body weight change, average daily weight gain (ADG), and feed conversion efficiency (FCE) of local sheep fed with varying quantities of DDML are shown in Table 2. The results revealed that the groups provided with DDLM supplements exhibited significantly (*p* < .05) higher average final body weight and daily growth compared to the control groups. The FCE of the supplemented groups was also notably greater than that of the control groups.

**TABLE 2:** The effect of supplementing different levels of dawri‐damaa (DDLM) on body weight change of the experimental sheep during a 90‐day feeding trial.

Parameter	Treatments				SEM	LS
	T_1_	T_2_	T_3_	T_4_		
IBW (kg)	23.77	23.52	23.72	23.85	0.73	Ns
FBW (kg)	25.12^d^	28.12^c^	29.83^b^	31.40^a^	0.89	***
BWC (kg)	1.35^d^	4.60^c^	6.12^b^	7.55^a^	0.56	***
ADG (g)	15.0^d^	51.40^c^	68.00^b^	83.80^a^	6.24	***
FCE	0.025^c^	0.066^b^	0.079^ab^	0.089^a^	0.007	***

Abbreviations: ADG, average daily gain; BWC, body weight change; FBW, final body weight; FCE, feed conversion efficiency; IBW, initial body weight; LS, level of significance; ns, nonsignificant; SEM, standard error of the mean; T_1_, hay ad libitum + 100 g ground maize; T_2_, T_1_ + 178 g DDLM; T_3_, T_1_ + 297 g DDLM; T_4_, T_1_ + 415 g DDLM.

^abcd^Mean values with different superscripts within a row are significantly different at *p* < .05; ***p < .05.

The ADG of the sheep given DDLM supplements ranged from 51.4 to 83.8 g/day, which was significantly (*p* < .05) higher than the control group's ADG of 15.0 g/day. This was consistent with the findings of Ajebu et al. (2018), Chala et al. (2019), and Tagaynesh (2014) for Horro sheep and Hararghe Highland sheep, respectively. The current study, on the other hand, reported larger body weight gain than prior studies, which found −22.1 to 25.3 g/day for Washera sheep by Gebru et al. (2015) and −14.67 to 33.22 g/day for Somali goats by Taddesse et al. (2014).

A positive ADG (15 g/day) in control animals could be attributable to grass hay and ground maize meeting the minimal need, even with slight development (McDonald et al., 2002). On the other hand, the sheep experienced superior BWC and ADG at T4. Furthermore, changes in BWC and ADG among treatments in the current investigation could be attributed to differences in daily DM consumption and feed digestibility (McDonald et al., 2011).

The FCE of supplemented animals ranged from 0.066 to 0.089 in the current study, which is comparable to the reports (0.06–0.13) by Kiflay et al. (2014) and Tagaynesh (2014). Feed conversion efficiency gains with DDLM supplementation were associated with increased CP intake and digestibility, suggesting that nutritional availability for growth in supplemented sheep was increased.

**FIGURE 1:**
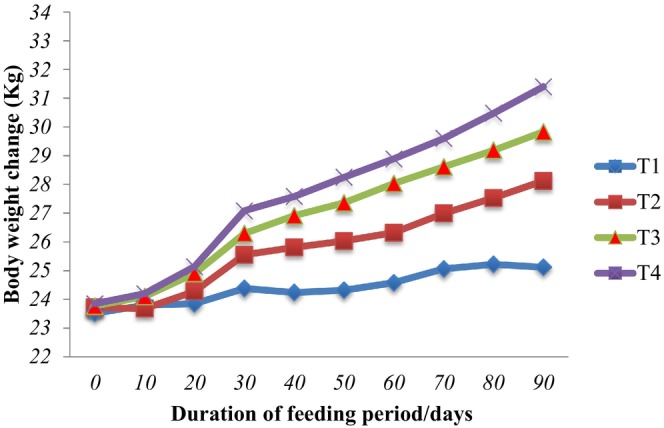
Trends in body weight changes of local growing lambs in Dawuro‐fed native grass hay supplemented with different levels of dawri‐damaa leaf meal during a 90‐day feeding period.

With the exception of the 40th and 90th days, the trend of weight changes across feeding trial days (Figure 1) demonstrated that all sheep across control treatments showed slight body weight gain up to the 90th day. This could be related to poor nutritional composition, specifically the lack of CP in the basal diet to promote optimal growth, as well as increased demand for nutrients for metabolic reactions. Those in the supplemented group, on the other hand, experienced continuous increases in body weight. Moreover, the rate of weight change in supplemented lambs (T4 > T3 > T2) might be related to the rate of increased CP and ME consumption and the trend of increasing digestibility coefficient. Nevertheless, all treatment levels improved FBW, ADG, and FCE in the current study, indicating that the comparative nutritional content of DDLM feed used in the current study could be supplemented to local lambs fed with poor quality feed resources.

**FIGURE 2:**
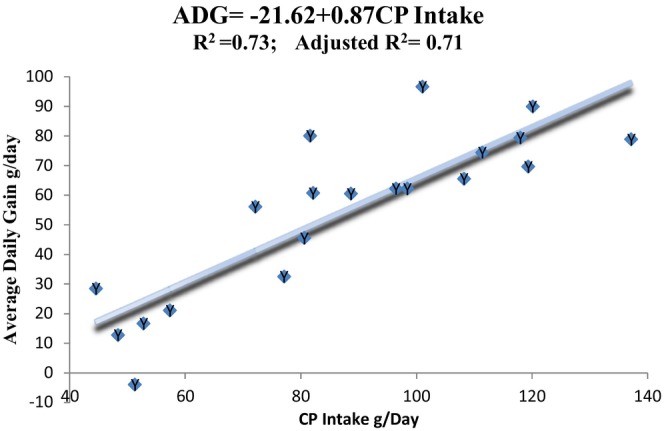
Regression of average daily gain (ADG) over crude protein (CP) intake of Dawuro local lambs fed native grass hay supplemented with different levels of dawri‐damaa leaf meal during a 90‐day feeding period.

Figure 2 depicts the effect of ADG on CP consumption of local lambs fed experimental diets. In fact, nutrient contribution was critical in lamb development parameters, while the influence of CP intake on daily live weight gain is significant. The current investigation found a positive and linear correlation (*p* < .05) between ADG and total CP intake (*r* ^2^ = 0.73). This shows that the average daily gain of lambs fed native grass hay with 100 g ground maize supplemented with varied amounts of DDLM is 73% dependent on total CP consumption. Abuye et al. (2018) found *r* ^2^ = 0.57, whereas Emebet (2008) and Dereje (2012) reported *r* ^2^ = 0.78 and 0.76, respectively, which are in line with the current study's findings. This study suggests that supplementing local lambs fed with poor quality feed resources with DDLM feed could significantly improve body weight, average daily gain, and feed conversion efficiency. These findings have implications for enhancing the nutritional content of sheep diets and promoting optimal growth in sheep production systems.

### 3.2 Characteristics of main carcass components

Table 3 displays the carcass characteristics of experimental lambs fed varied amounts of supplementary DDLM. The DDLM‐supplemented groups had significantly higher slaughter body weight (SBW), empty body weight (EBW), and hot carcass weight (HCW) than the control treatment groups (*p* < .05). Dressing percentage as a proportion of SBW (DPSBW) and dressing percentage as a proportion of EBW (DPEBW) increased with increasing DDLM supplementation, although this was not significant across treatments (*p* > .05). Furthermore, the rib‐eye muscle area (REMA) of the supplemented groups was significantly higher (*p* < .05) than the control groups, most likely due to lower total DM and nutrient intake, as well as poor apparent digestibility.

**TABLE 3:** Main carcass components of Dawuro local sheep fed grass hay and supplemented with different levels of dawri‐damaa leaf meal (DDLM).

Parameters	Treatments				SEM	LS
	T_1_	T_2_	T_3_	T_4_		
SBW (kg)	25.36^c^	28.50^b^	30.20^ab^	31.62^a^	0.88	***
EBW (kg)	20.20^c^	23.15^b^	24.72^ab^	25.53^a^	0.80	**
HCW (kg)	10.69^c^	12.50^b^	13.26^ab^	14.07^a^	0.57	*
REMA (cm^2^)	9.10^c^	12.30^b^	14.35^a^	14.20^a^	0.60	***
*Dressing percentage (DP)*						
DPSBW%	41.96	43.51	43.52	44.39	0.76	Ns
DPEBW%	52.71	53.52	53.24	55.03	0.77	Ns

Abbreviations: DPEBW, dressing percentage as a proportion to EBW; DPSBW, dressing percentage as a proportion to SBW; EBW, empty body weight; HCW, hot carcass weight; LS, level of significance; Ns, nonsignificant; REMA, rib‐eye muscle area; SBW, slaughter body weight; SEM, standard error of the mean; T_1_, hay ad libitum + 100 g ground maize; T_2_, T_1_ + 178 g DDLM; T_3_, T_1_ + 297 g DDLM; T_4_, T_1_ + 415 g DDLM.

^abc^Mean values with different superscripts within a row are significantly different at *p* < .05; *p < .05; **p < .01; ***p < .001.

When spineless cactus diets were substituted for sugarcane diets in lamb feed, de Oliveira et al. (2018) reported similar maximum values for SBW, HCW, and CCW. Energy intake has been proven in sheep to change tissue deposition, resulting in carcass gains (Pereira et al., 2010; Piola Junior et al., 2009). In the current study, sheep treated with DDLM had higher SBW and EBW, suggesting that DDLM supplementation can improve the nutritional content of low‐quality roughages.

According to Alexandre et al. (2008) and Salo et al. (2016), as slaughter body weight increased, so did HCW. Thus, the higher carcass production reported in supplemented animals is compatible with higher ADG, most likely due to the usage of forage legumes and the function of higher protein turnover in the muscle (Anderson et al., 2005).

As the rib‐eye muscle area (REMA) increases, so does the amount of muscle in the carcass, and the yield grade (USDA, 2009). A REMA at T1 was 9.1 cm^2^, and the undersupplemented diet was 12.3–14.2 cm^2^, which was greater than the 7.4–12.6 cm^2^ reported by Dejen (2011) and the 8.41–10.8 cm^2^ reported by Tekliye et al. (2018) for Farta sheep, as well as 11.20 cm^2^ for Santa Inês sheep fed Tifton grass hay and fresh spineless cactus (Andrade et al., 2016). In the current study, the varied levels of DDLM in the diet had a significant impact on REMA. de Oliveira et al. (2018) and Pereira et al. (2010) observed very identical average values of 12.90 cm^2^ for Santa Inês lambs fed spineless cactus as a replacement for sugarcane and 12.56 cm^2^ for Santa Inês sheep carcass. However, this value is lower than the average of 13–19.5 cm^2^ for Wogera sheep fed on natural grass hay and supplemented with a graded quantity of dry beer grain (Moges et al., 2008). The wide range of results could be attributed to a number of factors, including age, breed, kind of sheep, nutrition, and so on. According to McDonald et al. (2010), nutrition, age, sex, genetics, season, and other critical factors influence animal growth and carcass features.

On DPEBW and DPSBW, there was no significant difference (*p* > .05) between supplemented and nonsupplemented animals (Mechaele, 2018; Mengistu, 2016; Teklu et al., 2017; Wondimagegn, 2012). The dressing percentage is influenced by the animal's gut fill on an SBW and EBW basis. For example, animals in the control group had lower DPSBW% and DPEBW%, whereas animals in the supplemented groups had higher magnitudes due to the consequences of higher levels of nutrition and nutrient utilization in tissue development of the rams (Taylor & Murray, 1991) and breed of the rams (Devendra & Burns, 1983; McDonald et al., 2002).

The study found that supplementing sheep with DDLM resulted in increased body weight, average daily gain, and feed conversion rate. The three doses of Dawri‐damma supplementation (30%, 50%, and 70%) resulted in daily increases of 51.4 g, 68.0 g, and 83.8 g, respectively, which outperformed the national meat production average of 50.0 g per animal per day (Melaku & Betsha, 2008) and were significantly higher than those observed in lambs fed a basal diet of 15.0 g in this study. The carcass characteristics, including SBW, EBW, and HCW, were also significantly influenced for the DDLM‐supplemented groups compared to the control treatment.

This study demonstrates that adding shrubs, herbs, and legumes, such as DDLM, to a grazing system improves the body size and productivity of local sheep. The values obtained for the relevant variables were greater than the control diets for SBW, EBW, and HCW values, indicating that DDLM is a good supplement in some accessible locations that it is suited for sheep feed.

### 3.3 Edible offal

Table 4 shows the results of the influence of varied levels of DDLM supplementation on edible offal components of the experimental sheep. The study found that the kidney, tail, reticulo‐rumen, omasum, abomasum, small intestine, total fat, and bile were significantly different (*p* < .05) between the groups fed T1, T2, T3, and T4. However, the liver, heart, tongue, and large intestine were not significantly different (*p* > .05). The groups fed T2, T3, and T4 had no significant difference (*p* > .05) in the tail, omasum, abomasum, and small intestine compared to each other, but all were significantly higher than the group fed T1. The bulk of the edible and economically relevant offal components (TEOC) was significantly altered by DDLM supplementation (*p* < .05).

**TABLE 4:** Effect of different levels of dawri‐damaa leaf meal (DDLM) supplementation on edible offal components of local sheep fed native grass hay.

Parameters	Treatments				SEM	LS
	T_1_	T_2_	T_3_	T_4_		
Kidney (g)	83.4^b^	89.8^ab^	96.0^a^	97.0^a^	2.01	*
Heart (g)	145.0	120.2	122.2	122.0	4.317	ns
Liver (g)	397.2	388.4	419.8	428.2	9.72	Ns
Tongue (g)	80.4	83.4	82.2	96.8	2.98	Ns
Tail (g)	281.0^b^	585.4^a^	669.0^a^	608.4^a^	48.93	**
R_R (g)	626.6^ab^	571.8^b^	733.0^a^	725.6^a^	20.08	***
Omasum (g)	83.0^b^	117.6^ab^	113.2^a^	118.6^a^	4.99	**
Abomasum (g)	162.6^b^	223.2^a^	212.8^a^	218.8^a^	8.50	**
Small intestine (g)	606.4^b^	759.4^a^	767.6^a^	763.0^a^	24.30	***
Large intestine (g)	556.8	446.40	452.8	445.0	26.74	Ns
Kidney fat (g)	40.4^b^	57.4^ab^	68.0^ab^	87.0^a^	4.95	***
Heart fat (g)	35.2^b^	49.4^ab^	59.8^a^	64.0^a^	3.24	***
Bile (g)	23.4^b^	46.4^ab^	34.0^ab^	51.0^a^	3.04	***
Omental fat (g)	203.6^b^	212.6^ab^	239.6^ab^	257.2^a^	8.18	**
TEOC (kg)	3.33^b^	3.75^ab^	4.07^a^	4.08^a^	0.12	***

Abbreviations: LS, significant level; Ns, nonsignificant; R_R, reticulo‐rumen; SEM, standard error of the mean; T_1_, hay ad libitum + 100 g ground maize; T_2_, T_1_ + 178 g DDLM; T_3_, T_1_ + 297 g DDLM; T_4_, T_1_ + 415 g DDLM; TEOC, total edible offal content.

^abc^Mean values with the same superscripts within a row are not significantly different at (*p* < .05); ***Significant (*p* < .001); **Significant (*p* < .01); *Significant (*p* < .05).

According to Richardson et al. (2004), noncarcass components contribute 5% of the variation in feeding efficiency. The grand mean of total edible and economically relevant offal components (TEOC) for supplemented lambs in the current study ranged from 3.75 to 4.08 kg. This result is comparable to the value of 3.74–4.16 kg reported for Horro sheep (Chala et al., 2019), higher than the 3.2 kg reported for Tigray highland sheep supplemented with cottonseed meal (Amare et al., 2009), but lower than the 4.3–5.4 kg reported for Begait sheep fed grass hay and supplemented with various levels of concentrate mixture (Kibrom, 2018).

It is challenging to compare the TEOC of one breed/type to another because each breed has its own characteristic feature for corresponding metrics, which can be modified by age, sex, plane of nutrition, breed, and other associated factors. Furthermore, significant variances may be linked to the method of edible and nonedible noncarcass component classification, which is more or less subjective to one's tradition, beliefs, culture, and preferences (Getahun, 2001). In general, increasing the amount of DDLM added to the basal diet of native grass improved the total quantity of edible offal contents.

### 3.4 Nonedible offal

The effect of varying levels of DDLM supplementation on nonedible offal components of the experimental sheep is presented in Table 5. The majority of the nonedible carcass components were unaffected by the various doses of supplementation (*p* > .05). Giblets, such as lung with trachea, skin, feet, and gut content, on the other hand, responded positively (*p* < .05) to DDLM administration, with no significant difference between supplementation doses. In general, total nonedible giblet was significantly higher (*p* < .05) in groups fed varying levels of DDLM supplementation, consistent with the findings of Chala et al. (2019) for Horro sheep. Skin weight acquired from animals fed a control diet may be attributed to insufficient SBW, which is correlated with skin (Mohammed & Yagoub, 2016).

**TABLE 5:** Effect of different levels of dawri‐damaa leaf meal (DDLM) supplementation on nonedible offal components of local sheep fed native grass hay and ground maize.

Parameters (g)	Treatments				SEM	LS
	T_1_	T_2_	T_3_	T_4_		
Blood (g)	1249.4	1426.0	1270.6	1439.4	39.11	Ns
Head (g)	1615.6	1549.8	1688.0	1616.0	29.93	Ns
Lung with trachea	283.6^b^	356.0^a^	390.6^a^	400.0^a^	11.85	**
Testis (g)	286.6	344.6	330.6	297.2	11.58	Ns
Penis (g)	54.0	58.8	52.8	54.8	1.37	Ns
Skin (g)	2111.0^b^	2467.2^ab^	2898.4^a^	2809.0^a^	108.84	**
Spleen (g)	59.8	65.6	72.0	71.8	4.59	Ns
Gut content (g)	5169.8^c^	5321.0^bc^	5483.4^b^	6086.8^a^	105.20	*
Bladder (g)	18.4	24.4	19.8	22.6	1.06	Ns
Feet (g)	471.0^b^	547.6^ab^	604.8^a^	611.0^a^	18.12	***
TNOC (kg)	11.32^c^	12.16^bc^	12.81^ab^	13.41^a^	0.24	***

Abbreviations: LS, level of significance; ns, nonsignificant; SEM, standard error of the mean; T_1_, hay ad libitum + 100 g ground maize; T_2_, T_1_ + 178 g DDLM; T_3_, T_1_ + 297 g DDLM; T_4_, T_1_ + 415 g DDLM; TNOC, total non‐edible offal content.

^abc^Mean values with the same superscripts within a row are not significantly different at (*p* > .05); ***Significant at *p* < .001; **Significant (*p* < .01); *significant (*p* < .05).

There was no significant difference between all the treatment groups in mean weight of blood and spleen (*p* > .05). Because the spleen stores blood to release during stressful conditions, the volume of blood on the animal's body has a positive relationship with the size of the spleen.

Although not affected by supplementation, the decreased spleen size of the T1 groups could be attributed to a concurrent decline in blood weight. Treatment 1 had the lowest value for lung with trachea and feet, which could be related to the weight of sheep after slaughter. The higher subcutaneous layer fat storage of sheep may explain the difference in skin weight (Lawrence et al., 2012).

Unsupplemented lambs’ gut contents differed due to a longer retention time of ingested feed in the gastro‐intestinal tract (Pond et al., 1995), lower degradability and insufficient nutrient supply (Mahgoub et al., 2000), and a decrease in dietary energy level (Yagoub & Babiker, 2008). Gut content was also linked to total nonedible offal content (TNOC), which was lower in supplemented sheep than in control (T1) Begait sheep (Abraham, 2019).

### 3.5 Correlation between body weight gain and carcass parameters

The correlation between ADG, HCW, TEOC, and dressing percentage on the basis of slaughter and empty body weight (SBW and SBW) is presented in Table 6. Average daily gain was shown to be favorably associated to FCE and REMA (*p* < .001), as well as to HCW and TEOC (*p* < .05). Feed conversion efficiency had no relationship with carcass features (*p* > .05), although it was moderately and positively correlated with REMA (*p* > .05). It was discovered that HCW and the other carcass measures had a positive correlation. Tesfaye and Solomon (2008) found a favorable relationship between REMA, HCW, and dressing percentages (SBW and EBW) in Afar rams fed teff (*Eragrostis tef*) straw supplemented with graded degrees of concentrate mixtures. The REMA was found to be positively associated to HCW and TEOC.

**TABLE 6:** Correlation between body weight gain and carcass characteristics of the experimental sheep fed on different levels of supplementary dawri‐damaa leaf meal.

	ADG	FCE	HCW	TEOC	DPSBW	DPEBW
ADG						
FCE	0.94***					
HCW	0.54*	0.31 Ns				
TEOC	0.46*	0.19 Ns	0.71**			
DPSBW	0.38 ns	0.24 Ns	0.89***	0.35 Ns		
DPEBW	0.34 ns	0.21 Ns	0.83***	0.25 Ns	0.98***	
REMA	0.76***	0.56*	0.85***	0.80 ***	0.63**	0.54*

Abbreviations: ADG, average daily gain; DPEBW, dressing percentage as a proporation to EBW; DPSBW, dressing percentage as a proporation to SBW; FCE, feed conversion efficiency; HCW, hot carcass weight; ns, nonsignificant; REMA, rib‐eye muscle area; TEOC, total edible offal content.

***p < .001; **p < .01; *p < .05.

A research piece assessing the plant's nutritional value suggested *P. schimperiana's* potential supplementation as a protein source for animal feed (Kochare et al., 2023; Woretaw et al., 2022). Other researches also revealed that, in comparison to grazing lambs, lambs finished in a feedlot or with supplementation under extensive systems grow more quickly, reach target weights more rapidly, and produce larger carcass weights (De Brito et al., 2017; Jimenez et al., 2019).

### 3.6 Partial budget analysis

According to the study, the purchasing costs of lambs across each treatment were essentially identical, as indicated in Table 7. However, the cost of hay declined with increasing degree of supplementation while overall feed cost increased, so the total cost of production increased with increasing level of supplementation. Thus, the highest cost (ETB 437.61) was seen among the supplemented, and the best selling price (ETB 1634.61) was received for the lambs fed on T4. Similarly, the total return (TR) and net return (NR) were higher because the lambs were given the maximum level of supplement. This means that higher production costs may have resulted in a higher total return per animal. In general, the economic return of the trial was revealed to be mostly determined by feed expenses, purchasing and selling prices of the experimental sheep.

**TABLE 7:** Partial budget analysis of local sheep fed grass hay and supplemented with different levels of dawri‐damaa leaf meal (DDLM).

Variables	T_1_	T_2_	T_3_	T_4_
Purchasing price of sheep (ETB/head)	1010.31	999.77	1008.02	1013.62
Total hay consumed (kg/head)	47.86	43.67	41.90	39.60
Total DDLM consumed (kg/head)	–	16.72	28.03	37.55
Maize consumed (kg/head)	9	9	9	9
Cost of hay (ETB/head)	167.52	152.84	146.66	138.60
Cost of DDLM (ETB/head)	–	104.54	175.17	234.66
Cost of maize (ETB/head)	64.35	64.35	64.35	64.35
TVC (ETB/head)	231.87	321.73	386.19	437.61
Selling price of sheep (ETB/head)	1310.30	1474.96	1563.03	1634.61
TR (ETB/head)	299.99	475.26	555.01	620.99
NR (ETB/head)	68.12	153.53	168.82	183.38
∆TVC	–	89.86	154.32	205.74
∆NR	–	85.41	100.70	115.26
MRR%	–	95.05	65.25	56.02

Abbreviations: ∆NR, change in net return; ∆TVC, change in total variable cost; DDLM, dawri‐damaa leaf meal; ETB, Ethiopian birr; MRR, marginal rate of return; NR, net return; T_1_, hay ad libitum + 100 g ground maize; T_2_, T_1_ + 178 g DDLM; T_3_, T_1_ + 297 g DDLM; T_4_, T_1_ + 415 g DDLM; TR, total return; TVC, total variable cost.

In this study, the net return (NR) of sheep at T1 is ETB +68.12, indicating that no economic loss was seen when no DDLM was supplemented. The difference in price between control and treated lambs was attributed to differences in BWC of the lambs in each treatment, which was a function of feed quality differences and hence greater FCE. In general, lambs with higher nutrient intake had higher ADG, which resulted in a higher sales price and a larger net return.

The study found that the net return (NR) was larger (ETB 183.38) at the higher level (T4) in the supplemented group. The ∆NR was greatest (115.26) at the same level of supplementation. This suggests that the trial's best ∆NR value is at T4, but the higher marginal return appears at T2, because any marginal return is dependent on the same amount of marginal production cost (TVC). The marginal rate of return (MRR) demonstrated that each extra unit of 1 ETB per sheep cost increment resulted in marginal returns of 0.95, 0.65, and 0.56 ETB for T2, T3, and T4, respectively. As a result, the findings of this study revealed that supplementing DDLM with greater levels of T4 (415 g/day) was economically possible in light of its biological relevance.

The correlation between body weight gain and carcass parameters revealed favorable associations between average daily gain, carcass characteristics, and dressing percentage. Furthermore, a partial budget analysis demonstrated that higher levels of DDLM supplementation resulted in increased total return and net return, indicating the economic feasibility of supplementation.

## 4 CONCLUSIONS

In conclusion, the supplementation of dawri‐damaa leaf meal (DDLM) positively influenced sheep performance, carcass characteristics, and economic feasibility when fed native grass hay. The findings underscore the potential of DDLM as a valuable dietary supplement for improving sheep production and economic returns. However, economic analysis showed T2 as the optimal level of supplementation. Therefore, DDLM supplementation at 30% of daily DM intake on a 2.5% live weight basis can result in a higher marginal return. Considering the availability, access, and higher cost of compound feed, supplementing DDLM at 70% of their daily DM intake on the 2.5% live weight basis could be recommended to the producers.

It is suggested that further research be conducted to generate more information on the use of DDLM supplementation under farm conditions, using higher levels and different forms of preparation of the plant. Further research could also be done to evaluate the health of the animals.

## AUTHOR CONTRIBUTIONS

Gubil Bekele Dillo: Conceptualization (equal); data curation (equal); formal analysis (equal); investigation (equal); methodology (equal); resources (equal); software (equal); validation (equal); writing – original draft (equal); writing – review and editing (equal). Taye Tolemariam: Conceptualization (equal); methodology (equal); supervision (equal); visualization (equal); writing – original draft (equal); writing – review and editing (equal). Asrat Guja Amejo: Conceptualization (equal); methodology (equal); supervision (equal); visualization (equal); writing – original draft (equal); writing – review and editing (equal).

## CONFLICT OF INTEREST STATEMENT

The authors declare no competing interest.

## ETHICS STATEMENT

The study work was done according to ethical clearance made by the Research and Ethical Review Board/Institutional Review Board (RERB/IRB) of JUCAVM (Jimma University College of Agriculture and Veterinary Medicine). The Ethical Board has confirmed that feeding, handling, slaughtering, and data collection followed the standard procedures of the University adopted from Article 6 (Methods of killing) of Directive, 2010/63/EU of the European Parliament and of the Council of 22 September 2010 on the protection of animals used for scientific purposes.

## Data Availability

The corresponding author will provide the data used and analyzed for this study upon reasonable request.

## References

[fsn34156-bib-0001] Abera, M. , Tolera, A. , Nurfeta, A. , & Geleti, D. (2021). The effect of supplementation of vetch (Vicia villosa) on performance of Arsi‐bale sheep fed basal diet of Desho (*Pennisetum pedicellatum*) grass. Acta Agriculturae Scandinavica Section A Animal Science, 70(3–4), 123–131. 10.1080/09064702.2021.1976264

[fsn34156-bib-0002] Abraham, T. (2019). Feed intake, digestibility and growth performance of Begait sheep fed Hay as a basal diet and supplemented with *Tsara* (*Pterocarpus lucens*), pigeon pea (*Cajanes cajan*) leaves and concentrate mixture. International Journal of Livestock Production, 10(9), 204–212.

[fsn34156-bib-0003] Abuye, T. , Yadav, R. K. , & Diriba, C. (2018). Supplementary value of two *Lablab purpureus* cultivars and concentrate mixture to natural grass hay basal diet based on feed intake, digestibility, growth performance and net return of Horro sheep. International Journal of Livestock Production, 9(6), 140–150.

[fsn34156-bib-0004] Ajebu, N. , Diriba, C. , Banerjee, S. , & Lars, E. (2018). Effects of feeding different proportions of silver leaf desmodium (*Desmodium uncinatum*) with banana (*Musa paradisiaca*) leaf on nutrient utilization in Horro sheep fed a basal diet of natural grass hay. Asian‐Australasian Journal of Animal Sciences, 31, 1449–1457. 29514444 10.5713/ajas.17.0831 PMC6127577

[fsn34156-bib-0005] Alexandre, G. , Coppry, O. , Bocage, J. , & Archimède, H. (2008). Effect of live weight at slaughter on the carcass characteristics of intensively fattened Martinik sheep fed sugar cane supplemented with pea flour. LRRD, 20, 119.

[fsn34156-bib-0006] Amare, D. , Melaku, S. , & Berhane, G. (2009). Supplementation of iso‐nitrogenous oil seed cakes in cactus (*Opuntia ficus‐indica*)–*teff* straw (*Eragrostis tef*) based feeding of Tigray Highland sheep. Animal Feed Science and Technology, 148(2–4), 214–226.

[fsn34156-bib-0007] Amare, Y. E. , Geletu, M. , Dires, K. , Desta, K. , Ayalew, A. , Tilahun, A. , & Gashu, M. (2021). Evaluation of antidiabetic and antidyslipidemic effects of methanolic extract of pentas Schimperiana leaf in mice. Berhan International Research Journal of Science and Humanities, 5(1), 166–182.

[fsn34156-bib-0008] Andarge, E. , Shonga, A. , Agize, M. , & Tora, A. (2015). Utilization and conservation of medicinal plants and their associated indigenous knowledge (IK) in Dawuro zone: An ethnobotanical approach. International Journal of Medicinal Plant Research, 4, 330–337.

[fsn34156-bib-0009] Anderson, H. J. , Oksbjerg, N. , Young, J. F. , & Therkildsen, M. (2005). Feeding and meat quality – A future approach. Meat Science, 70(3), 543–554. 22063752 10.1016/j.meatsci.2004.07.015

[fsn34156-bib-0010] Andrade, S. F. J. D. , Batista, Â. M. V. , Carvalho, F. F. R. D. , Lucena, R. B. D. , Andrade, R. D. P. X. D. , & Lima Júnior, D. M. D. (2016). Fresh or dehydrated spineless cactus in diets for lambs. Acta Scientiarum. Animal Sciences, 38, 155–161.

[fsn34156-bib-0011] Animut, G. , & Wamatu, J. (2014). Prospects to improve the productivity of sheep fattening in Ethiopia: Status, challenges and opportunities. CARDA.

[fsn34156-bib-0012] ARC (Agricultural Research Council) . (1980). The nutrient requirements of ruminant live stock. Technical review by an Agricultural Research Council working party published on behalf of the Agricultural Research Council by the common wealth agricultural bureau (pp. 114–151). Famhan Royal.

[fsn34156-bib-0013] Calnan, H. B. , Jacob, R. H. , Pethick, D. W. , & Gardner, G. E. (2014). Factors affecting the color of lamb meat from the longissimus muscle during display: The influence of muscle weight and muscle oxidative capacity. Journal of Meat Science, 96(2), 1049–1057. 24080243 10.1016/j.meatsci.2013.08.032

[fsn34156-bib-0014] Chala, D. , Mengistu, U. , & Ulfina, G. (2019). Effect of replacement of concentrate with *Lablab purpureus* hay on feed intake, digestibility, live weight gain and carcass characteristics of Horro sheep fed natural pasture hay as basal diet. Global Scientific Journal, 7(11), 597–614.

[fsn34156-bib-0015] Chikwanha, O. C. , Vahmani, P. , Muchenje, V. , Dugan, M. E. , & Mapiye, C. (2018). Nutritional enhancement of sheep meat fatty acid profile for human health and wellbeing. Food Research International, 104, 25–38. 29433780 10.1016/j.foodres.2017.05.005

[fsn34156-bib-0016] De Brito, G. F. , Ponnampalam, E. N. , & Hopkins, D. L. (2017). The effect of extensive feeding systems on growth rate, carcass traits, and meat quality of finishing lambs. Comprehensive Reviews in Food Science and Food Safety, 16(1), 23–38. 33371548 10.1111/1541-4337.12230

[fsn34156-bib-0017] de Oliveira, J. P. F. , de Andrade Ferreira, M. , Alves, A. M. S. V. , de Melo, A. C. C. , de Andrade, I. B. , Urbano, S. A. , Suassuna, J. M. A. , de Barros, L. J. A. , & de Barros Melo, T. T. (2018). Carcass characteristics of lambs fed spineless cactus as a replacement for sugarcane. Asian‐Australasian Journal of Animal Sciences, 31(4), 529–536. 28823123 10.5713/ajas.17.0375 PMC5838325

[fsn34156-bib-0018] Dejen, M. (2011). *Effect of supplementation of hay with graded level of rapeseed cake and rice bran mixture on feed intake, digestibility, body weight change and carcass characteristics of Farta sheep*. Doctoral Dissertation, Haramaya University, Ethiopia.

[fsn34156-bib-0019] Delgado, C. L. (2003). Rising consumption of meat and milk in developing countries has created a new food revolution. The Journal of Nutrition, 133(11), 3907S–3910S. 14672289 10.1093/jn/133.11.3907S

[fsn34156-bib-0020] Dereje, K. (2012). *Evaluation of multi‐nutrient blocks and activated effective microorganisms on intake, digestibility, body weight changes and carcass parameters of intact Horro rams fed Rhodes grass hay*. A MSc. Thesis, Haramaya University, Dire Dawa, Ethiopia.

[fsn34156-bib-0021] Devendra, C. , & Burns, M. (1983). Goat production in the tropics: Commonwealth agriculture Bureaux (pp. 51–57). Farnham Royal Bucks.

[fsn34156-bib-0022] Dinku, T. , Tadesse, S. , & Asres, K. (2010). Antidiabetic activity of the leaf extracts of *Pentas schimperiana* subsp. schimperiana (A. Rich) Vatke on alloxan induced diabetic mice. Ethiopian Pharmaceutical Journal, 28(1), 22–26.

[fsn34156-bib-0023] Directive . (2010). Directive 2010/63/EU of the European Parliament and the Council of 22 September 2010 on the protection of animals used for scientific purposes. Official Journal of the European Union, 276, 33–79.

[fsn34156-bib-0024] Emebet, L. (2008). *Supplementation of blackhead Ogaden sheep fed haricot bean (Phaseolus vulgaris) haulms with mixtures of wheat bran and brewers dried grain: Effects on feed utilization, live weight gain and carcass parameters*. M.Sc. Thesis, Haramaya University Ethiopia, 39p.

[fsn34156-bib-0025] Erasmus, S. W. , Muller, M. , & Hoffman, L. C. (2017). Authentic sheep meat in the European Union: Factors influencing and validating its unique meat quality. Journal of the Science of Food and Agriculture, 97(7), 1979–1996. 27976419 10.1002/jsfa.8180

[fsn34156-bib-0026] FAO (Food and Agricultural Organization) . (2011). World livestock 2011 – Livestock in food security. FAO.

[fsn34156-bib-0027] FAO (Food and Agricultural Organization) . (2013). Current worldwide annual meat consumption per capita. FAO (food and agriculture Organization of the United Nations).

[fsn34156-bib-0028] Fitwi, M. , & Tadesse, G. (2013). Effect of sesame cake supplementation on feed intake, body weight gain, feed conversion efficiency and carcass parameters in the ration of sheep fed on wheat bran and teff (Eragrostis teff) straw. Momona Ethiopian Journal of Science, 5(1), 89–106. 10.4314/mejs.v5i1.85333

[fsn34156-bib-0029] Gebrekidan, G. , Teklebrhan, T. , & Tesfay, Z. (2019). Growth performance and carcass traits of begait lambs fed diets of cowpea (*Vigna unguiculata*) hay, wheat bran and their mixtures. Journal of Agriculture and Ecology Research International, 20(1), 1–12. 10.9734/JAERI/2019/v20i130097

[fsn34156-bib-0030] Gebru, T. , Firew, T. , Yeshambel, M. , Solomon, M. , & Atsushi, T. (2015). Effects of different forms of white lupin (*Lupinus albus*) grain supplementation on feed intake, digestibility, growth performance and carcass characteristics of Washera sheep fed Rhodes grass (*Chloris gayana)* hay‐based diets. Tropical Animal Health and Production, 47(8), 1581–1590. 26250152 10.1007/s11250-015-0901-9

[fsn34156-bib-0031] Getahun, K. (2015). Optimum dietary crude protein level for fattening yearling Arsi bale lambs. World Journal of Agricultural Sciences, 11(2), 101–106.

[fsn34156-bib-0032] Getahun, L. (2001). *Growth pattern and carcass characteristics of Somali and Mid‐rift Valley goats*. MSc Thesis, Haramaya University, Dire Dawa, Ethiopia.

[fsn34156-bib-0033] Gilmour, A. R. , Cullis, J. J. , Fogarty, N. M. , & Banks, R. (1994). Genetic parameter for ultrasonic fat depth and eye muscle measurement in live poll Dorset sheep. Australian Journal of Agricultural Research, 45, 1281–1291.

[fsn34156-bib-0034] Gurgeira, D. N. , Crisóstomo, C. , Sartori, L. V. C. , de Paz, C. C. P. , Delmilho, G. , Chay‐Canul, A. J. , Bedoya, H. J. N. , Vega, W. H. O. , Bueno, M. S. , & da Costa, R. L. D. (2022). Characteristics of growth, carcass and meat quality of sheep with different feed efficiency phenotypes. Meat Science, 194, 108959. 36084489 10.1016/j.meatsci.2022.108959

[fsn34156-bib-0036] Heba‐tollah, M. S. , Abd‐Alla, H. I. , Abdelwahab, A. B. , Gabr, M. M. , & Kirsch, G. (2018). Secondary metabolites and biological activity of pentas species: A minireview. Journal of Advanced Research, 10, 21–30. 30046473 10.1016/j.jare.2017.12.003 PMC6057236

[fsn34156-bib-0037] Henchion, M. , McCarthy, M. , Resconi, V. C. , & Troy, D. (2014). Meat consumption: Trends and quality matters. Meat Science, 98(3), 561–568. 25060586 10.1016/j.meatsci.2014.06.007

[fsn34156-bib-0038] Hou, Y. , Liu, C. , Su, L. , Zhao, L. , Yang, Z. , Bai, Y. , Dou, L. , Yao, D. , & Jin, Y. (2023). Dietary linseed supplementation improves meat quality and flavor of sheep by altering muscle fiber characteristics and antioxidant capacity. Animal Science Journal, 94(1), e13801. 36606309 10.1111/asj.13801

[fsn34156-bib-0040] Jimenez, L. E. R. , Naranjo, A. , Hernandez, J. C. A. , Ovalos, J. O. , Ortega, O. C. , & Ronquillo, M. G. (2019). A meta‐analysis on the effect of the feeding type and production system on the carcase quality of lambs. Italian Journal of Animal Science, 18(1), 423–434.

[fsn34156-bib-0041] Kibrom, G. (2018). *Effect of increasing level of supplementation on feed intake, nutrient utilization, growth and carcass characteristics of Begait sheep fed a basal diet of grass Hay*. MSc. Thesis, Haramaya University, Ethiopia.

[fsn34156-bib-0042] Kiflay, W. , Getachew, A. , & Mengistu, U. (2014). Effect of different levels of soybean*/glycine max/*meal supplementation on feed intake, digestibility, live weight changes, and carcass characteristics of black head Ogaden sheep. East African Journal of Sciences, 8(2), 135–146.

[fsn34156-bib-0043] Kifle, Z. D. , Abdelwuhab, M. , Melak, A. D. , Meseret, T. , & Adugna, M. (2022). Pharmacological evaluation of medicinal plants with antidiabetic activities in Ethiopia: A review. Metabolism Open, 13, 100174. 35296054 10.1016/j.metop.2022.100174 PMC8919291

[fsn34156-bib-0044] Kochare, T. , Delilo, G. , & Lendado, B. (2023). Carcass characteristics and economic analysis of supplementing powdered coffee leaves to indigenous Wolaita highland sheep diets based on hay. Cogent Food & Agriculture, 9(2), 2285227.

[fsn34156-bib-0045] Lage, R. R. P. , Vega, W. H. O. , Costa, H. H. A. , Costa, A. C. , Sousa, L. C. O. , Lima, L. D. , & Landim, A. V. (2020). Effect of breed on meat quality and global acceptance of native lambs and their crosses. South African Journal of Animal Science, 50(1), 150–160.

[fsn34156-bib-0082] Lawrence, T. , Fowler, V. , & Novakofski, J. (2012). Growth of farm animals. Cabi.

[fsn34156-bib-0046] Luo, Y. , Wang, B. , Liu, C. , Su, R. , Hou, Y. , Yao, D. , Zhao, L. , Su, L. , & Jin, Y. (2019). Meat quality, fatty acids, volatile compounds, and antioxidant properties of lambs fed pasture versus mixed diet. Food Science & Nutrition, 7(9), 2796–2805. 31572572 10.1002/fsn3.1039 PMC6766570

[fsn34156-bib-0047] Mahgoub, O. , Lu, C. D. , & Early, R. J. (2000). Effects of dietary energy density on feed intake, body weight gain and carcass chemical composition of Omani growing lambs. Small Ruminant Research, 37(1–2), 35–42. 10818301 10.1016/s0921-4488(99)00132-7

[fsn34156-bib-0048] McDonald, P. R. A. , Edwards, R. A. , Greenhalgh, J. F. D. , & Morgan, C. A. (2002). Animal nutrition (6th ed., pp. 245–477). Prentice Hall.

[fsn34156-bib-0049] McDonald, P. R. A. , Edwards, R. A. , Greenhalgh, J. F. D. , Morgan, C. A. , Sinclair, L. , & Wilkinson, R. (2011). Nutrient requirements of the lactating ewe. In Animal Nutrition (7th ed., pp. 444–449). Pearson Education Limited.

[fsn34156-bib-0050] McDonald, P. R. A. , Edwards, R. A. , Greenhalgh, J. F. D. , Morgan, C. A. , Sinclair, L. A. , & Wilkinson, R. G. (2010). Animal Nutrition (7th ed., p. 692). Pearson Education Limited.

[fsn34156-bib-0051] Mechaele, Y. (2018). *Effects of feeding dried acacia (saligna, Sesbania sesban OR Vigna unguiculata) leaves as: A replacement for cotton seed cake on production performances and semen quality of Begait sheep in northwestern Tigray, Ethiopia*. A Ph.D Dissertation, Addis Ababa University, Ethiopia.

[fsn34156-bib-0052] Mekonnen, K. , Gebreyes, M. , Abdulkadir, B. , Seifu, H. , & Thorne, P. J. (2021). Training module on livestock feed and forage innovations . https://cgspace.cgiar.org/bitstream/handle/10568/117289/Training_module_2021.pdf

[fsn34156-bib-0053] Melaku, S. , & Betsha, S. (2008). Bodyweight and carcass characteristics of Somali goats fed hay supplemented with graded levels of peanut cake and wheat bran mixture. Tropical Animal Health and Production, 40, 553–560. 18716913 10.1007/s11250-008-9133-6

[fsn34156-bib-0054] Mengistu, M. (2016). *Effect of graded levels of tagasaste (Chamaecytisus palmensis) leaf supplementation on performance of yearling Menz sheep in Ethiopian highlands*. PhD dissertation, Hawassa University, Ethiopia.

[fsn34156-bib-0055] Merhun, L. , Mengistu, U. , & Yoseph, M. (2016). Effects of supplementation with different levels of wheat bran and noug seed (*Guizotia abissynica*) cake mixtures on performance of Hararghe Highland sheep fed a basal diet of maize stover. American Journal of Agricultural Science, 30, 40–47.

[fsn34156-bib-0056] Moges, M. , Tamir, B. , & Yami, A. (2008). The effects of supplementation of grass hay with different levels of brewer s dried grain on feed intake digestibility and body weight gain in intact Wogera lambs. East African Journal of Sciences, 2(2), 105–110.

[fsn34156-bib-0057] Mohammed, A. H. , & Yagoub, Y. M. (2016). Effect of concentrate supplementation on the performance and carcass characteristics of natural grazing Sudanese Desert lambs. Journal of Agricultural Science and Engineering, 2(1), 1–4.

[fsn34156-bib-0058] NRC (National Research Council) . (1996). Nutrient requirement of sheep (Sixth revised ed.). National Academy press.

[fsn34156-bib-0059] OEC (Observatory of Economic Complexity) . (2021). Sheep and Goat meat in Ethiopia . Retrieved from https://oec.world/en/profile/bilateral‐product/sheep‐and‐goat‐meat/reporter/eth

[fsn34156-bib-0060] Pereira, E. S. , Pimentel, P. G. , Fontenele, R. M. , de Medeiros, A. N. , Regadas Filho, J. G. L. , & Villarroel, A. B. S. (2010). Characteristics and yields of carcass and cuts in Santa Inês sheep fed with different concentrations of metabolizable energy. Acta Scientiarum‐Animal Sciences, 32(4), 431–437.

[fsn34156-bib-0061] Piola Junior, W. , Ribeiro, E. L. D. A. , Mizubuti, I. Y. , Silva, L. D. D. F. D. , Sousa, C. L. D. , & Paiva, F. H. P. D. (2009). Levels of energy in the feeding of feedlot lambs and the regional and tissue carcass composition. Revista Brasileira de Zootecnia, 38, 1797–1802.

[fsn34156-bib-0062] Pond, W. G. , Church, D. C. , & Pond, K. R. (1995). Basic animal nutrition and feeding (4th ed., p. 601). John Wiley and Sons.

[fsn34156-bib-0063] Richardson, E. C. , Herd, R. M. , Archer, J. A. , & Arthur, P. F. (2004). Metabolic differences in Angus steers divergently selected for residual feed intake. Australian Journal of Experimental Agriculture, 44(5), 441–452.

[fsn34156-bib-0064] Salami, S. A. , Luciano, G. , O'Grady, M. N. , Biondi, L. , Newbold, C. J. , Kerry, J. P. , & Priolo, A. (2019). Sustainability of feeding plant by‐products: A review of the implications for ruminant meat production. Animal Feed Science and Technology, 251, 37–55.

[fsn34156-bib-0065] Salo, S. , Mengistu, U. , & Getachew, A. (2016). Effects of supplementation with different forms of barley on feed intake, digestibility, live weight change and carcass characteristics of Hararghe Highland sheep fed natural pasture. Journal of Food Processing and Technology, 7(3), 568.

[fsn34156-bib-0066] Taddesse, D. , Melaku, S. , & Mekasha, Y. (2014). Effect of supplementation of cactus and selected browses mix on feed utilization of Somali goats. American Scientific Research Journal for Engineering, Technology, and Sciences (ASRJETS), 9(1), 20–34.

[fsn34156-bib-0067] Tagaynesh, A. (2014). *Supplementation of sole wheat bran or its mixture with safflower seed cake on performance of Hararghe highland sheep fed grass hay basal diet*; A MSc. Thesis, Haramaya University, Dire Dawa, Ethiopia.

[fsn34156-bib-0068] Taylor, S. C. S. , & Murray, J. I. (1991). Effect of feeding level, breed and milking potential on body tissues and organs of mature, non‐lactating cows. Animal Science, 53, 27–38.

[fsn34156-bib-0069] Tekliye, L. , Mekuriaw, Y. , Asmare, B. , & Mehret, F. (2018). Nutrient intake, digestibility, growth performance and carcass characteristics of Farta sheep fed urea‐treated rice straw supplemented with graded levels of dried Sesbania sesban leaves. Agriculture & Food Security, 7, 1–10.

[fsn34156-bib-0070] Teklu, W. , Adugna, T. , Jane, W. , Getachew, A. , & Rischkowsky, B. (2017). Effects of feeding different varieties of faba bean (*Vicia faba* L.) straws with concentrate on feed intake, digestibility, and body weight gain and carcass characteristics of Arsi‐Bale sheep. Asian‐Australasian Journal of Animal Sciences, 31(8), 1221. 29268588 10.5713/ajas.17.0736 PMC6043437

[fsn34156-bib-0071] Tesfaye, H. , & Solomon, M. (2008). Feed intake, digestibility, body wight and carcass parameter of Afar rams fed *teff* (*Eragrostis tef*) straw supplemented with graded levels of concentrate mixtures. Tropical Animal Health and Production, 41(4), 599–606. 18777140 10.1007/s11250-008-9230-6

[fsn34156-bib-0072] Tikam, K. , Phatsara, C. , Sorachakula, C. , Vearasilp, T. , Samiprem, S. , Cherdthong, A. , Gerlach, K. , & Südekum, K. H. (2015). In vitro gas production, in vivo nutrient digestibilities, and metabolisable energy concentrations for sheep of fresh and conserved pangola grass. Small Ruminant Research, 128, 34–40.

[fsn34156-bib-0073] Tonamo, A. , Tamir, B. , & Goshu, G. (2015). Assessment of cattle feed resources; chemical composition and digestibility of major feeds in Essera district, Southern Ethiopia. Science, Technology and Arts Research Journal, 4(2), 89–98. 10.4314/star.v4i2.12

[fsn34156-bib-0074] Upton, M. (1979). Farm Management in Africa: The principle of production and planning (pp. 282–298). Oxford University Press.

[fsn34156-bib-0075] USAID (United States Agency for International Development) . (2021). Feed the future Ethiopia value chain activity: partnering with agricultural growth program meat and live animal market price brief 01 . https://pdf.usaid.gov/pdf_docs/PA00Z883.pdf

[fsn34156-bib-0076] USDA (The United State Department of Agriculture) . (2009). *Beef grades and carcass information. M Enterprises Ltd*., USA. Production (ESAP) held in Addis Ababa, Ethiopia, 27–29 April 1995.

[fsn34156-bib-0077] Wang, B. , Yang, L. , Luo, Y. , Su, R. , Su, L. , Zhao, L. , & Jin, Y. E. (2018). Effects of feeding regimens on meat quality, fatty acid composition and metabolism as related to gene expression in Chinese Sunit sheep. Small Ruminant Research, 169, 127–133.

[fsn34156-bib-0078] Williams, A. R. (2002). Ultrasound applications in beef cattle carcass research and management. Journal of Animal Science, 80(E‐suppl_2), E183–E188.

[fsn34156-bib-0079] Wondimagegn, B. (2012). *Effects of feeding different levels of detoxified Ethiopian mustard (Brassica carinata) seed cake on live weight gain and carcass parameters of Afar sheep fed native hay basal diet*; A MSc. Thesis, Haramaya University, Ethiopia.

[fsn34156-bib-0080] Woretaw, T. , Beyero, N. , & Kassu, Y. (2022). Pentas schimperiana utilization practices as livestock feed in Mareka District, Ethiopia. Advances in Animal and Veterinary Sciences, 10(2), 277–285.

[fsn34156-bib-0081] Yagoub, Y. M. , & Babiker, S. A. (2008). Effect of dietary energy level on growth and carcass characteristics of female goats in Sudan. Livestock Research for Rural Development, 20(12), 2008.

